# Association between rs3087243 and rs231775 polymorphism within the cytotoxic T-lymphocyte antigen 4 gene and Graves' disease: a case/control study combined with meta-analyses

**DOI:** 10.18632/oncotarget.22702

**Published:** 2017-11-27

**Authors:** Yaqin Tu, Guorun Fan, Yu Dai, Tianshu Zeng, Fei Xiao, Lulu Chen, Wen Kong

**Affiliations:** ^1^ Department of Otorhinolaryngology, Union Hospital, Tongji Medical College, Huazhong University of Science and Technology, Wuhan 430022, China; ^2^ Department of Endocrinology, Union Hospital, Tongji Medical College, Huazhong University of Science and Technology, Wuhan 430022, China; ^3^ Department of Nuclear Medicine, Union Hospital, Tongji Medical College, Huazhong University of Science and Technology, Wuhan 430022, China

**Keywords:** Graves’ disease, cytotoxic T-lymphocyte antigen 4, polymorphism, susceptibility, case/control study

## Abstract

We conducted a case/control study to assess the impact of SNP rs3087243 and rs231775 within the *CTLA4* gene, on the susceptibility to Graves' disease (GD) in a Chinese Han dataset (271 cases and 298 controls). The frequency of G allele for rs3087243 and rs231775 was observed to be significantly higher in subjects with GD than in control subjects (*p* = 0.005 and *p* = 0.000, respectively). After logistic regression analysis, a significant association was detected between SNP rs3087243 and GD in the additive and recessive models. Similarly, association for the SNP rs231775 could also be detected in the additive model, dominant model and recessive model. A meta-analysis, including 27 published datasets along with the current dataset, was performed to further confirm the association. Consistent with our case/control results, rs3087243 and rs231775 showed a significant association with GD in all genetic models. Of note, ethnic stratification revealed that these two SNPs were associated with susceptibility to GD in populations of both Asian and European descent. In conclusion, our data support that the rs3087243 and rs231775 polymorphisms within the *CTLA4* gene confer genetic susceptibility to GD.

## INTRODUCTION

Graves' disease (GD) is an autoimmune thyroid disease with a 0.5% rate of prevalence in the general population [1]. It is characterized by the presence of thyroid-stimulating hormone (TSH) receptor antibodies leading to hyperthyroidism and goiter. The exact etiology of GD remains unknown; however, it is believed that genetic polymorphisms and environmental factors are both involved in pathogenesis. It has now been established that the thyroid gland in patients with GD is infiltrated by lymphocytes, predominantly Τ lymphocytes; a T lymphocyte immune regulating gene cluster is located in the 2q33 gene region [2–3]. The cytotoxic T-lymphocyte antigen 4 (*CTLA4*) gene, residing in human chromosome 2q33, encodes a key negative regulator of T-cell activation and proliferation during the immune response and thereby may influence T-cell mediated autoimmune diseases such as GD [4–5]. Therefore, the *CTLA4* gene is a functional candidate genetic marker for studying GD.

**Figure 1:**
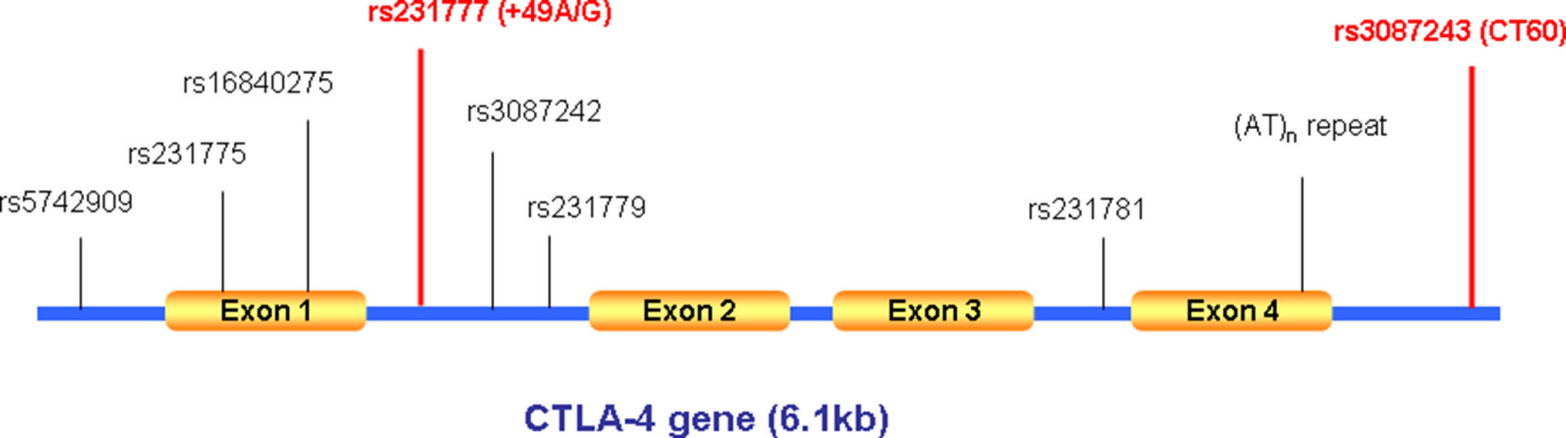
The human *CTLA4* gene structure and known polymorphisms

Full-length human *CTLA4* gene spans 6.1kb DNA, with 4 exons and 3 introns. This small region is very polymorphic as manifested by the enrichment of many exonic, intronic, and promoter single nucleotide polymorphisms (SNPs). Although the mechanisms underlying CTLA4 mediated GD development are yet to be fully addressed, elucidation of its genetic predisposition to GD, however, may offer some important clues. Indeed, several SNPs within the *CTLA4* gene (C/T polymorphism in the promoter region -318, A/G polymorphism in exon 1 +49 (rs231775), microsatellite (AT)_n_ repeat in the 3′-UTR of exon 4, and CT60 (rs3087243) in the 6.1-kb 3′ noncoding region (Figure 1) have been suggested to be associated with the development of GD by several genome-wide association studies (GWAS) [6–8]. Nevertheless, the results are somehow inconsistent between the different populations studied. Interestingly, there is plausible evidence indicating that the A to G substitution in exon 1 (rs231775) is possibly linked with T-lymphocyte activation, and the A to G substitution in 3′ noncoding region (rs3087243) is possibly associated with the splicing and production efficiency of soluble CTLA4, and indeed, more data consistent with this association have been reported for these two SNPs, especially in Asian populations [9–11]. We, therefore, conducted a case/control study together with meta-analysis by employing, to our knowledge, all published eligible case/control datasets. We demonstrated convincing evidence supporting the association of rs3087243 and rs231775 polymorphism with increased risk of GD.

## RESULTS

### Clinical and biochemical features of the study subjects

Clinical and biochemical features of the study subjects are summarized in Table 1. In total, 271 patients with GD and 298 ethnically and geographically matched controls were included in this study. No significant differences were observed between patients with GD and control subjects in terms of sex, while the average age of patients with GD was significantly lesser than that of control subjects. However, patients with GD manifested significantly higher levels of FT3, FT4, and TRAB and lower levels of TSH compared to that of control subjects (Table 1).

**Table 1:** Clinical characteristics for the study subjects

Clinical characteristic	GD	Control	*P* value
Number	271	298	/
Sex (% male)	25.1%	22.8%	0.53
Age (years)	37.3 ± 15.0	41.4 ± 14.7	0.001
FT3 (pmol/ml)	17.0 ± 13.2	4.9 ± 0.7	*p* < 0.001
FT4 (pmol/ml)	47.5 ± 27.1	17.4 ± 2.1	*p* < 0.001
TSH (mIU/L)	0.2 ± 1.0	1.9 ± 0.9	*p* < 0.001
TRAB (IU/L)	12.1 ± 11.3	0.5 ± 0.3	*p* < 0.001

Data are shown as mean ± standard deviation.

FT3: free triiodothyronine; FT4: free thyroxine; TSH: Thyroid Stimulating Hormone; TRAB: TSH Receptor Antibodies; GD: Graves' disease

### Results for case/control study

The genotyping results for all patients and controls are summarized in Table 2. The genotypic distribution of SNP rs3087243 and rs231775 was found to be in Hardy-Weinberg equilibrium for the control subjects (*p* > 0.05). Interestingly, the frequency for G allele both in rs3087243 and rs231775 was observed to be significantly higher in subjects with GD than in control subjects (*p* = 0.005 and *p* = 0.000, respectively). After logistic regression analysis, a significant association between SNP rs3087243 and the risk of GD was detected in the additive model (OR = 1.50, 95% CI, 1.12 – 2.00, *p* = 0.007) and recessive model (OR = 1.70, 95% CI, 1.19 – 2.41, *p* = 0.004). Similarly, association for the SNP rs231775 could also be detected in the additive model (OR = 2.66, 95% CI, 2.02 – 3.50, *p* = 0.000), dominant model (OR = 3.47, 95% CI, 1.91–6.30, *p* = 0.000) and recessive model (OR = 3.57, 95% CI, 2.51–5.07, *p* = 0.000) (*p* < 0.025 was considered with statistical significance after Bonferroni correction, Table 2).

**Table 2:** Results for case/control study in the Chinese Han population

SNP	Genotypes		Alleles			*P*_additive_	*P*_dominant_	*P*_recessive_
rs3087243	A/A	GG+AG	A	G	*P*-value			
GD	12	259	89	453	**0.005**	**0.007**	**0.329**	**0.004**
Control	18	280	139	457				
rs231775	A/A	GG+AG	A	G				
GD	16	255	107	435	**0.000**	**0.000**	**0.000**	**0.000**
Control	50	248	241	355				

*P*-values were obtained by logistic analyses of three alternative models (additive, dominant, and recessive model) through adjusting for age and gender. *P* < 0.025 was considered with statistical significance after Bonferroni correction.

### Identification of eligible datasets for meta-analysis

**Figure 2:**
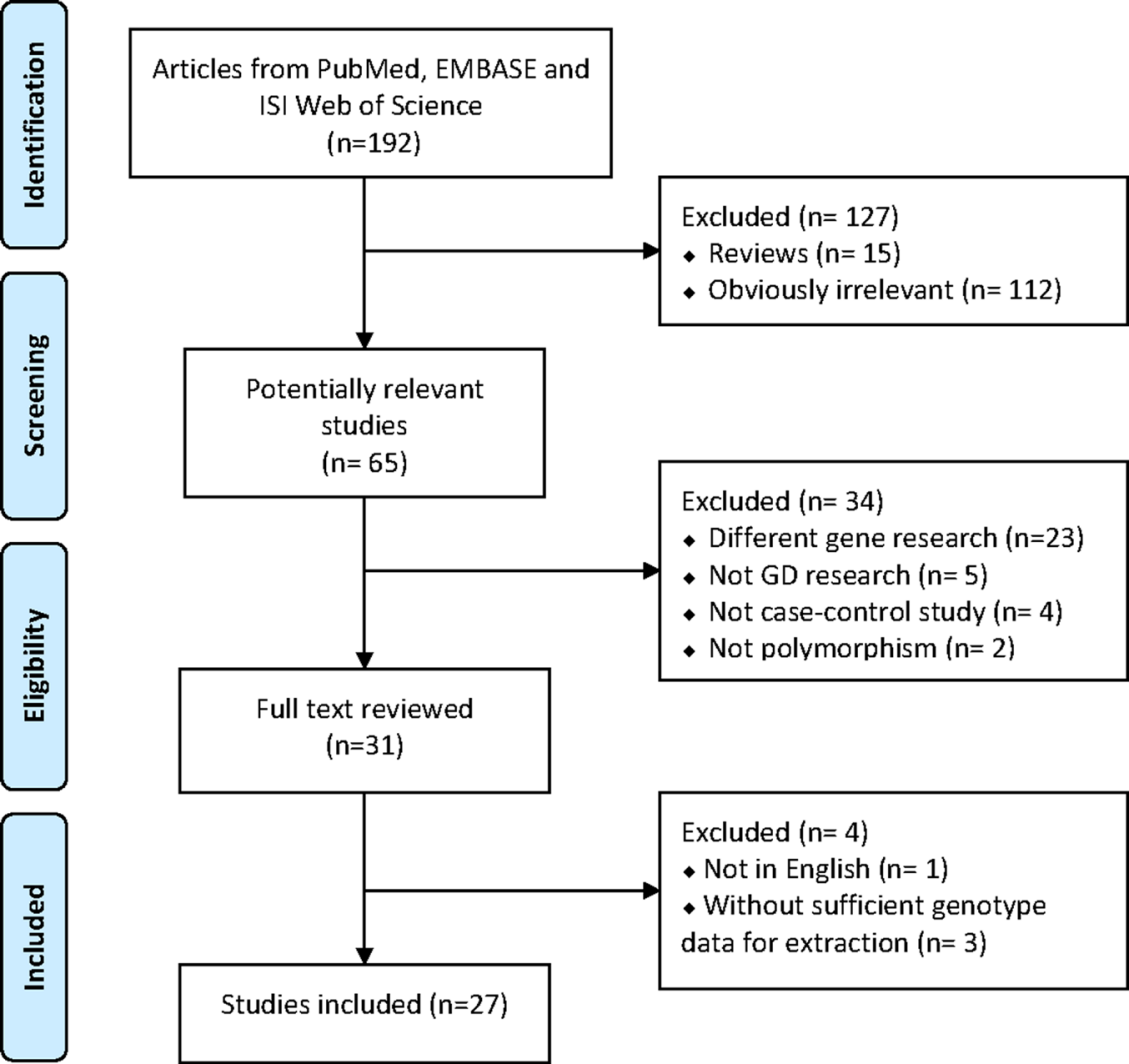
Flow chart for selection of eligible datasets for systematic reviews and meta-analysis (PRISMA)

To further demonstrate the association between the above SNP and GD risk, we next sought to conduct a meta-analysis. In total, 192 publications were relevant to the search words, of which 112 studies were obviously irrelevant, and 15 articles were unacceptable since they were reviews. Additionally, 34 studies were excluded because twenty-three of the articles focused on different genes, another 11 were also excluded because they were not on GD research (5 studies), not a case-control study (4 studies) or polymorphism (2 articles). Among the remaining 31 publications, 4 studies were rejected because they either did not present detailed genotyping information (3 article) or were published in non-English journals (1 study) (Figure 2).

### Characteristics for the selected datasets

In total, 27 case/control studies were identified based on our selecting criteria. Of which, 13 studies were conducted for the rs3087243 polymorphism which included 2299 type 2 diabetic patients and 4176 controls, while 22 studies were carried out for the rs231775 polymorphism which involved 5001 patients and 5774 controls. The principal characteristics and genotype distributions of the identified studies are shown in Table 3. For SNP rs3087243 polymorphism, ten studies came from East Asian [12–21] and three studies from European population [22–24]. For the rs231775 polymorphism, there were 14 studies originated from East Asian [13, 17–21, 25–32], while the rest 8 studies were from European population [6, 23–24, 33–37]. Genotypic distribution for both rs3087243 and rs231775 in controls was in consistent with HWE (*p* > 0.05) except for the 4 datasets highlighted in boldface font in the Table 3.

**Table 3:** Summary of datasets included for meta-analysis

ID	Author	Year	Ethnicity	Genotyping method	Study design	SNP loc	Case/Control	GD genotype			Control genotype			*p*HWE
								AA	AG	GG	AA	AG	GG	
1	Heward et al.	1999	European	PCR-RFLP	CC	rs231775	379/363	122	192	65	164	171	28	0.07
2	Park et al.	2000	Asian	PCR-RFLP	CC	rs231775	97/199	5	35	57	26	75	98	0.06
3	Kouki et al.	2000	European	PCR-RFLP	CC	rs231775	45/43	8	29	8	15	23	5	0.39
4	Hadj et al.	2001	European	PCR-RFLP	CC	rs231775	144/205	31	63	50	26	94	85	0.99
5	Kouki et al.	2002	Asian	PCR-RFLP	CC	rs231775	120/80	22	67	31	30	36	14	0.58
6	Yung et al.	2002	Asian	SSCP	CC	rs231775	123/158	3	54	66	23	36	14	0.99
7	Vaidya et al.	2003	European	PCR-RFLP	CC	rs231775	301/349	88	139	74	146	158	45	0.83
8	Kalantari et al.	2003	Asian	PCR-RFLP	CC	rs231775	90/90	21	49	20	30	53	7	**0.013**
9	Petrone et al.	2005	European	Taqman	CC	rs3087243	150/301	26	79	45	83	149	69	0.89
						rs231775		59	68	23	139	138	24	0.201
10	Weng et al.	2005	Asian	PCR-RFLP	CC	rs3087243	107/101	2	27	78	2	45	54	**0.033**
						rs231775	104/101	5	53	46	15	58	28	0.091
11	Ban et al.	2005	Asian	PCR-RFLP	CC	rs3087243	131/179	2	38	91	14	70	95	0.83
12	Cho et al.	2006	Asian	Sequencing	CC	rs3087243	283/472	7	78	198	13	125	334	0.75
						rs231775	288/471	16	112	160	30	197	244	0.24
13	Han et al.	2006	Asian	DCFH	CC	rs3087243	261/196	8	71	182	13	60	123	0.14
						rs231775	261/196	32	94	135	32	89	75	0.52
14	Wang et al.	2007	Asian	PCR-RFLP	CC	rs3087243	189/192	5	46	138	9	58	125	0.50
						rs231775	208/192	15	69	124	18	77	97	0.63
15	Chong et al.	2008	Asian	Taqman	CC	rs3087243	177/151	4	48	125	12	51	88	0.24
						rs231775		7	73	97	24	56	71	**0.03**
16	Tsai et al.	2008	Asian	PCR-RFLP	CC	rs3087243	189/192	5	48	136	9	58	125	0.50
17	Namo et al.	2008	European	PCR-RFLP	CC	rs231775	217/149	116	43	58	78	39	32	**0.000**
18	Sahin et al.	2009	Asian	PCR-RFLP	CC	rs231775	77/98	29	33	15	43	48	7	0.19
19	Esteghamati et al.	2009	Asian	PCR-RFLP	CC	rs231775	205/103	114	71	20	75	25	3	0.61
20	Kimura et al.	2009	Asian	PCR-RFLP	CC	rs231775	415/795	62	143	210	142	358	295	0.07
21	Bicek et al.	2009	European	Taqman	CC	rs3087243	123/117	16	57	50	26	61	30	0.63
						rs231775		33	73	17	47	64	6	0.01
22	Kimkong et al.	2011	Asian	PCR-RFLP	CC	rs3087243	132/153	8	46	78	12	59	82	0.76
23	Yang et al.	2012	Asian	PCR-RFLP	CC	rs231775	303/215	12	139	152	29	97	89	0.75
24	Jurecka-Lubieniecka	2013	European	PCR-RFLP	CC	rs231775	620/340	159	302	159	126	156	58	0.42
25	Chen et al.	2015	Asian	PCR-HRM	CC	rs3087243	260/248	20	100	140	40	101	107	0.056
26	Pawlak-Adamska et al.	2016	European	PCR-RFLP	CC	rs3087243	172/388	18	80	74	68	187	133	0.87
27	Ting et al.	2016	Asian	PCR-RFLP	CC	rs3087243	554/1058	10	149	395	46	382	630	0.21
						rs231775		34	209	311	112	469	477	0.84
Our study	Tu et al.	2017	Asian	Sequencing	CC	rs3087243	271/298	12	65	194	18	103	177	0.56
						rs231775	271/298	16	75	180	50	141	107	0.76

CC: case/control

HWE: Hardy-Weinberg equilibrium

DCFH: Dual-color fluorescence hybridization technique

PCR-HRM: Polymerase chain reaction-high resolution melting

### Results of the SNP rs3087243 meta-analysis

**Figure 3:**
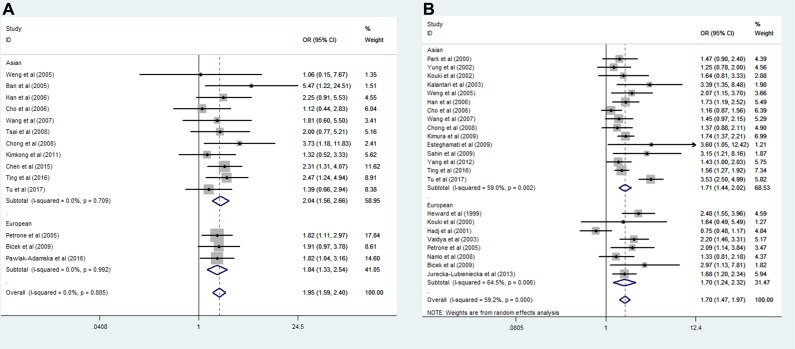
Forest plot for the association of CTLA4 rs3087243 and rs231775 polymorphism with Graves' disease after ethnic stratification (**A**) dominant model for SNP rs3087243 [(GG + GA) vs. AA)], (**B**) recessive model for SNP rs231775 [GG vs. (GA + AA)].

Meta-analysis for the SNP rs3087243 was performed by the above identified 13 datasets and our current dataset (2999 cases and 4474 controls in total). Significant association were found in the homozygote model(GG vs. AA: OR = 2.27, 95% CI = 1.83–2.82, *p* = 0.000), heterozygote model (GA vs. AA: OR = 1.61, 95% CI = 1.30–2.00, *p* = 0.000), dominant model (GG + GA vs. AA: OR = 1.95, 95% CI = 1.60–2.40, *p* = 0.000), recessive model (GG vs. GA+ AA: OR = 1.54, 95% CI = 1.40–1.71, *p* = 0.000) and additive model (G vs. A: OR =1.49, 95% CI = 1.37–1.61, *p* = 0.000) (Table 4). For analysis of ethnic stratification, we divided the datasets into 2 subgroups, East Asians and European. Given that no significant genetic heterogeneity was noted, a fixed effects model was thus employed for the analysis. GD susceptibility was significantly detected both in the Asian and European descent populations in all genetic models (Figure 3).

**Table 4:** Results for meta-analysis of CTLA4 polymorphisms with Graves' disease risk

SNPs	OR(95% CI)	*p* value	Test of heterogeneity		*p* for publication bias^a^
			I^2^	*p* value	
rs3087243 (A > G)					
GG vs. AA	2.27 [1.83, 2.82]	**0.000**	0.0%	0.822	0.779
GA vs. AA	1.61 [1.30, 2.00]	**0.000**	0.0%	0.931	0.675
GG + GA vs. AA	1.95 [1.60, 2.40]	**0.000**	0.0%	0.885	0.753
GG vs. GA + AA	1.54 [1.40, 1.71]	**0.000**	14.5%	0.294	0.543
G vs. A allele	1.49 [1.37, 1.61]	**0.000**	11.7%	0.325	0.601
rs231775 (A > G)					
GG vs. AA	2.41 [1.91, 3.05]	**0.000**	64.0%	0.000	0.058
GA vs. AA	1.46 [1.22, 1.74]	**0.000**	56.9%	0.000	0.084
GG + GA vs. AA	1.73 [1.45, 2.06]	**0.000**	61.1%	0.000	0.072
GG vs. GA + AA	1.70 [1.47, 1.97]	**0.000**	59.2%	0.000	0.251
G vs. A allele	1.50 [1.36, 1.65]	**0.000**	61.9%	0.000	0.687

^a^Egger's test was performed to assess publication bias.

### Results of the SNP rs231775 meta-analysis

Next, we conducted a meta-analysis for SNP rs231775 by including 6,115 cases patients and 7,155 controls. A significant association between rs231775 and GD risk was characterized in the homozygote model (GG vs. AA: OR = 2.41, 95% CI = 1.91–3.05, *p* = 0.000), heterozygote model (GA vs. AA: OR = 1.61, 95% CI = 1.22–1.74, *p* = 0.000), dominant model (GG + GA vs. AA: OR = 1.95, 95% CI = 1.45–2.06, *p* = 0.000), recessive model (GG vs. GA+ AA: OR = 1.54, 95% CI = 1.47–1.97, *p* = 0.000) and additive model (G vs. A: OR = 1.49, 95% CI = 1.36–1.65, *p* = 0.000) (Table 4). Analysis of ethnic stratification revealed that the association between rs231775 and GD tended to be much stronger for East Asian than European in all genetic models (Figure 3). Of note, our meta-analysis for SNP rs231775 was hampered by the presence of genetic heterogeneity, which could be due to the differences of ethnicities and gene-environmental interactions.

### Publication bias

**Figure 4:**
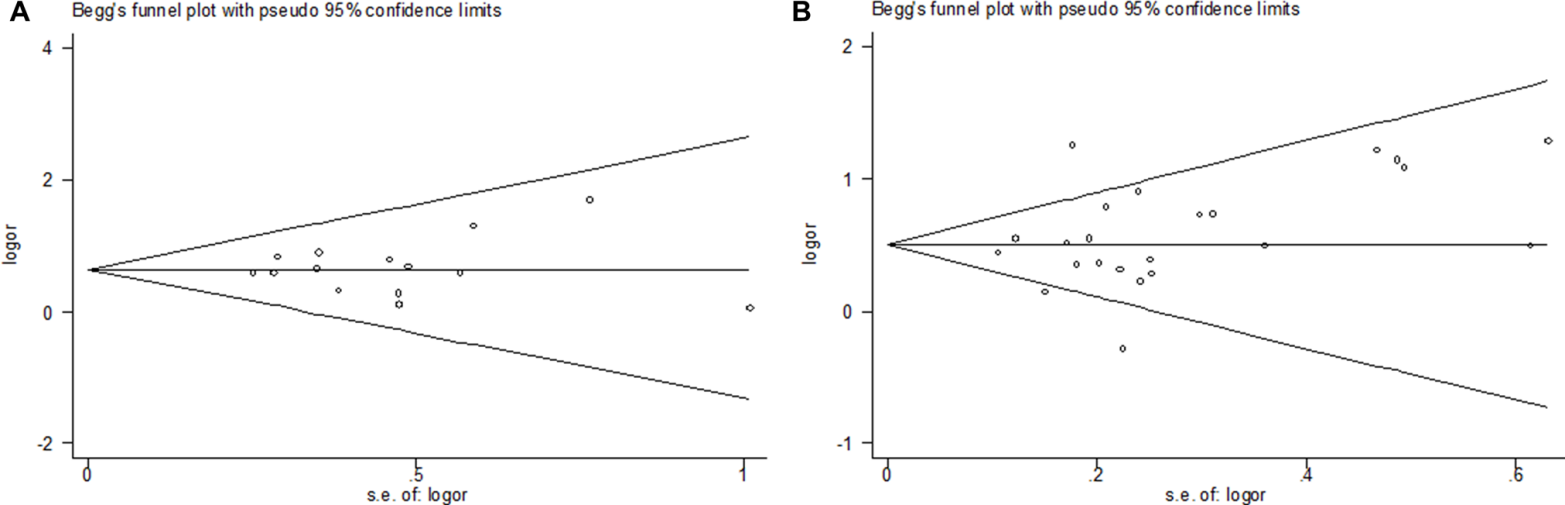
Funnel plot analysis to detect publication bias Each point represents a separate study for the indicated association. (**A**) Begg's funnel of publication bias test for SNP rs3087243: [(GG+GA) vs. AA)]; (**B**) Begg's funnel of publication bias test for SNP rs231775: [GG vs. (GA+AA)].

Begg's funnel plot and Egger's test were performed to assess publication bias. The shape of the funnel plots appeared to be symmetrical [SNP rs3087243: (GG + GA) vs. AA; SNP rs231775: GG vs. (GA+ AA)] and the Egger's test did not show any evidence of publication bias (Figure 4). Analysis of sensitivity also revealed that results derived from our study are stable and reliable (data not shown).

## DISCUSSION

Cytotoxic T-lymphocyte antigen 4 encoded by the *CTLA4 gene* is involved in controlling the proliferation and apoptosis of T lymphocytes, which is an essential contributing factor for the development of GD. Since the recognition of this functional property of *CTLA4*, it has been considered a candidate gene for GD. Nevertheless, thus far, no consistent results have been reported in terms of its genetic predisposition in GD pathoetiology. To address this issue, we conducted a case/control study focusing on the two SNPs, rs3087243 located in 3′ UTR and rs231775 located in exon 1 within the *CTLA4* gene, which included 271 patients with GD and 298 matched controls. Meta-analysis using the 27 published datasets along with our current study dataset was performed next to confirm the association further. Collectively, our study reported convincing evidence that SNP rs3087243 and rs231775 within *CTLA4* predisposes subjects with an increased risk of developing GD.

In our case/control study, the G allele frequency for both rs3087243 and rs231775 was observed to be significantly higher in patients with GD than in the control subjects suggesting that the G allele may confer an increased risk of GD. Indeed, a significant association was detected between the G allele of rs3087243 and susceptibility to GD in the additive and recessive models. Similarly, association for the SNP rs231775 could also be detected in the additive model, dominant model and recessive model after Bonferroni correction. Of note, there was a significant difference in terms of average age between patients with GD and control subjects in our dataset. This discrepancy is most likely caused by the impact of age on the risk of GD. The impact of genetic factors may be higher than environmental factors in patients with young age at GD diagnosis. Many studies have reported that GD occurs more frequently in young female patients. For example, Wang and colleagues reported that the average age of patients GD was significantly lesser than that of the control group [18]. Given that aging may induce reduction in the presence or intensity of signs and symptoms of the disease, it could be more difficult to diagnose hyperthyroidism [38]. Consistent with our observations, similar differences in average ages were also noted in other published datasets [18, 20, 39].

Our subsequent meta-analysis using 27 published datasets and our current study dataset provided additional evidence for the association between the two SNPs (rs3087243 and rs231775) and the risk of GD. To determine the influence of population stratification, we then divided all the datasets into two subgroups, East Asian and European descent. We found that rs3087243 and rs231775 polymorphism were associated with susceptibility to GD in both the Asian and European descent populations in all genetic models. These results were consistent with those reported by Chong et al. and Ting et al. from two other independent Chinese Han datasets. However, Kimkong and his colleagues failed to detect the association between CTLA4 CT60 polymorphism and GD in Thai population [40]. Given that factors such as living style and diet, social and emotional stress, as well as medical care and economic conditions are likely to be very diverse between each population group, heterogeneity could therefore be present even within the same ethnic group [41]. Of course, other factors such as study design and limited sample size may also contribute to the study discrepancies.

SNP rs3087243 is located downstream of the poly (A) termination site. Previous studies have suggested that this polymorphism is important for efficient splicing and production of soluble CTLA4 (sCTLA4), and may play a role in the mRNA stability of sCTLA4. In line with this notion, subjects with the putative risk G/G genotype have lower production of sCTLA4 than that by subjects with the protective A/A genotype [11]. The soluble form of CTLA4 is translated and secreted in human serum and can bind to CD80/86 molecules. Recombinant sCTLA4 inhibits T-cell proliferation *in vitro*, indicating that a reduction in the levels of this form of CTLA4 could lead to inefficient blocking of the immune response, triggering an autoimmune disorder [42]. The exonic SNP rs231775 in the CTLA4 leader peptide results in threonine to alanine change, and researchers found that the polymorphism decreased CTLA4 cell surface expression and reduced the inhibitory function of CTLA4, and the G allele reduced the ability to control T-cell proliferation [43–45].Therefore, this might confer a lesser CTLA4 function, resulting in greater T-cell activity, stronger immune response, and a higher probability of autoimmunity.

Despite that we employed a comprehensive analytic strategy to conduct our current studies; some issues are probably still needed to be pointed out. First, although our systematic meta-analysis with sufficient statistical power demonstrated a consistent conclusion as our case/control data, the sample size for our case/control dataset was relatively small. Second, the cases were significantly younger than the controls. It is merely due to selection bias. Considering this bias would lead to false positivity or false negativity, we will explore the association between these polymorphism and GD after age stratification in the future study. Third, we do not have enough data for assessing the differences of gene-environment interactions. Therefore, additional studies with a large dataset along with informative data for assessing the differences of gene - environmental interactions would be necessary.

In summary, our case/control study combined with meta-analysis provided convincing evidence that the CTLA4 polymorphisms confer genetic susceptibility to GD in the Chinese Han population. Specifically, the rs3087243 and rs231775 within the *CTLA4* gene are more susceptible to the genetic predisposition of GD. Future studies in a large dataset and focused on addressing the functional relevance of this polymorphism within the *CTLA4* gene would be necessary for fully establishing the impact on susceptibility to GD.

## MATERIALS AND METHODS

### Study subjects

We studied 271 Chinese patients with GD (37.3 ± 15.0 years old, 68 males and 203 females) recruited from the department of Endocrinology and 298 ethnically and geographically matched controls (41.4 ± 14.7 years old, 68 males and 230 females) from the Physical examination center, Union Hospital of Huazhong, University of Science and Technology. GD was diagnosed according to the clinical and laboratory features including (1) Biochemical assessment of hyperthyroidism (raised serum fT3 or fT4 levels, suppressed serum thyrotropin: TSH levels); (2) diffuse enlargement of thyroid gland; (3) presence of antithyroglobulin antibodies; and (4) other related symptoms such as exophthalmos and pretibial myxedema. The combination of diffuse goiter and prolonged hyperthyroidism is nearly always caused by Graves' disease, and the presence of characteristic eye or skin changes is diagnostic. Documentation of elevated TRAB can confirm the diagnosis. The normal reference range of indicators were as follows: TSH: 0.27–4.2 mIU/L; free T4 (fT4): 12–22 pmol/L; and free T3 (fT3): 3.1–6.8 pmol/L; TRAB: 0–1.75 IU/L. Control subjects with a family history of type 1 diabetes, autoimmune thyroid disease, or any other autoimmune diseases were excluded. All participants provided written informed consent. This study complied with the principles of the Declaration of Helsinki and was approved by the Institutional Review Board (IRB) and the ethics committees of the Union Hospital of Huazhong, University of Science and Technology.

### DNA isolation and genotyping

**Figure 5:**
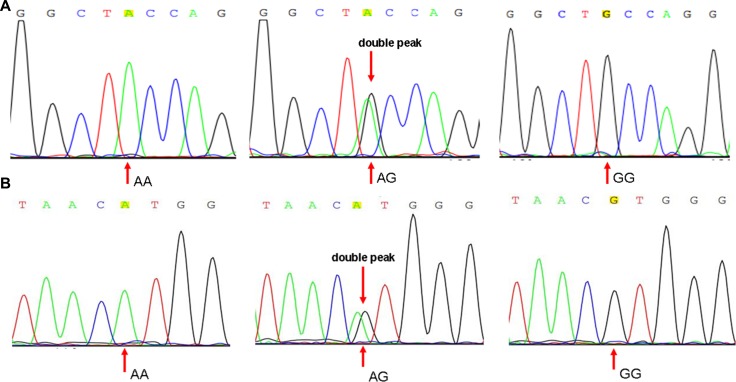
Sequence diagrams of SNP rs3087243 and rs231775 (**A**) Sequence diagrams of rs231775; (**B**) Sequence diagrams of rs3087243.

Genomic DNA was extracted from 1ml of peripheral blood sample of each subject using a genomic DNA extraction kit (TianGen, China) and according to the manufacturer's instructions. Genotyping of the rs3087243 and rs231775 polymorphism was performed by direct sequencing with an ABI 3730XL genetic analyzer (Applied Biosystems, Foster City, CA, USA) (Figure 5). These primers are as follows:

rs3087243: Forward - CTT CAT GAG TCA GCT TTG CAC CAG C

Reverse - AGC TGA GAA AGC AGG CGG TAA GAA A

rs231775: Forward - GCT CTA CTT CCT GAA GAC CT

Reverse - AGT CTC ACT CAC CTT TGC AG

### Thyroid function tests and determination of autoantibodies

The serum concentrations of FT3 (Roche Diagnostics GmbH, Mannheim, Germany), FT4 (Roche Diagnostics GmbH, Mannheim, Germany), TSH (Roche Diagnostics GmbH, Mannheim, Germany), and TRAB(Roche Diagnostics GmbH, Mannheim, Germany) were measured by commercial kits and Roche cobas e601 fully automated immunochemistry analyzer.

### Statistical analysis

Comparison of biochemical data between GD patients and control subjects was carried out by *t* tests. The Pearson's *χ*^2^ test was used to analyze the frequencies of genotypes between cases and controls. Hardy–Weinberg equilibrium (HWE) was evaluated by the goodness-of-fit *χ*^2^ test for genotypes in the control group. Unconditional multivariate logistic regression analysis was used to estimate odds ratios (ORs) and their 95% confidence intervals (CIs) after adjusting for sex and age. The statistical power of the study was calculated using PowerV3.0 software (http://www.mds.qmw.ac.uk/statgen/dcurtis/software.html). For SNPs with minor allele frequency (MAF) of 0.2, the power of our sample size was calculated to be 0.943 for detecting an OR of 1.91 in the case control study. This assumption on an OR was based on two different studies, which conducted on the Taiwan population and Slovenian population (OR of 1.91 and 2.0, respectively). All those statistical analyses were performed by means of the SPSS 18.0 software (SPSS Inc. Chicago, IL, USA). For the purpose of correcting for multiple testing, Bonferroni correction was applied. Consequently, differences were considered significant when the *p* value was less than 0.025.

### Meta analysis

PubMed, EMBASE and ISI Web of Science were searched (the last search was conduct on March 1, 2017) using the following search terms: “CTLA4” or “Cytotoxic T-Lymphocyte-Associated Antigen 4” and “Graves' disease” and “polymorphism”. Reference, which were listed in each identified article, were also searched manually to identify additional eligible studies. To be eligible, the following inclusion criteria were established: (1) a human case–control study of a polymorphism associated with Graves' disease; (2) studies that included sufficient genotype data for extraction. Main exclusion criteria of studies were as follows: (1) case reports, letters, reviews, and editorial articles; (2) literature not containing information regarding Graves' disease research; (3) study involving only a case population; and (4) study not written in English. In the case of multiple studies by the same researchers involving the same or overlapping data sets, we selected the most recent study with the largest number of participants.

Two curators (Yaqin Tu and Guorun Fan) independently extracted information from included studies. Disagreement was resolved by discussion between the two authors. The following data were extracted: first author's name, year of publication, the ethnicities of the individuals involved, the genotyping method, number of cases and controls for each genotype, and the Hardy-Weinberg equilibrium (HWE) among the controls. Ethnicity was categorized as East Asian and European. A double-check procedure was performed to ensure accuracy of data entry.

The strength of associations between SNP rs3087243 and rs231775 within the CTLA4 gene and Graves' disease risk was measured by ORs with 95% CIs. We explored the association between rs3087243 and Graves' disease in homozygote model (GG vs. AA), heterozygote model (GA vs. AA), dominant model [(GG + GA) vs. AA)], recessive model [GG vs. (GA + AA)] and additive model (G vs. A), respectively. The same genetic models were applied for SNP rs231775 as well. The Chi-squared-based Q-statistic test was used to assess the between-study heterogeneity, and it was considered significant if *P* < 0.10. When the effects were assumed to be homogenous, fixed-effects model was used (the Mantel–Haenszel method); otherwise, it was more appropriate to use random-effects model (DerSimonian and Laird method). Sensitivity analysis was performed to assess the influence of each individual study by omitting 1 study at a time and calculating a pooled estimate for the remainder of the studies. The inverted funnel plots and Egger's regression test were used to investigate publication bias. Potential publication bias was assessed with funnel plots of the effect sizes versus the standard errors; Begg's test was used to identify significant asymmetry. If there is evidence of publication bias, funnel plot is noticeably asymmetric. Concerning the significance level of the Begg's and Egger's tests was set at 0.05. All statistical tests carried out in the present report were two tailed and all analyses were conducted using the STATA 11.0 software (STATA Corporation, College Station, TX, USA).

## Abbreviations

GD: Graves' disease
CTLA4: Cytotoxic T-lymphocyte antigen 4
TSH: thyroid-stimulating hormone
GWAS: genome-wide association studies
CI: confidence interval
OR: odds ratio
SNPs: single nucleotide polymorphisms
HWE: Hardy-Weinberg equilibrium
TRAB: TSH Receptor Antibodies
